# High-Level Production of NMN in *Escherichia coli* Through Co-Utilization of Glucose and Glycerol

**DOI:** 10.3390/microorganisms14040897

**Published:** 2026-04-16

**Authors:** Jiajia Gan, Xiuzhen Chen, Yongzhi He, Yanfeng Zhang, Jin Zhong, Zhiyang Dong

**Affiliations:** 1 State Key Laboratory of Microbial Diversity and Innovative Utilization, Institute of Microbiology, Chinese Academy of Sciences, Beijing 100101, China; ganjiajia@im.ac.cn (J.G.); chenxiuzhen@im.ac.cn (X.C.); heyz@im.ac.cn (Y.H.); 2 School of Life Science, University of Chinese Academy of Sciences, Beijing 100049, China; 3 Shenzhen Siyomicro Bio-Tech Co., Ltd., Shenzhen 518100, China; zhangyf@siyobio.com

**Keywords:** nicotinamide mononucleotide, phosphoribosyl pyrophosphate, glucose–glycerol co-utilization, *Escherichia coli*, microbial cell factory

## Abstract

Nicotinamide mononucleotide (NMN), a direct precursor of the essential coenzyme nicotinamide adenine dinucleotide (NAD^+^), confers anti-aging effects and multiple health benefits. Engineered microorganisms represent a promising platform for sustainable industrial production of NMN. Here, the previously reported NMN-producing strain NMN008 was engineered to co-utilize glucose and glycerol for the biosynthesis of NMN from nicotinamide (NAM). First, the glycolytic genes *pgi* and *pykA*/*pykF* were sequentially deleted to disrupt glucose catabolism through the glycolytic pathway, thereby potentially improving precursor availability for NMN biosynthesis. Second, a feedback-resistant glycerol kinase mutant (glpK*) was introduced to enhance glycerol utilization, aiming to compensate for the growth defects associated with impaired glycolysis. These modifications enabled glycerol to primarily support cell growth and energy metabolism, while improving glucose allocation toward NMN biosynthesis by reducing its competitive consumption through glycolysis. As a result, the final strain achieved an NMN titer of 32.92 g/L in a 2 L bioreactor, representing a 26.28% increase in NMN production and a substantial 34.48% improvement in carbon conversion efficiency. Our research provides an effective strategy to achieve industrial-scale production of NMN, laying a foundation for the widespread application of NMN.

## 1. Introduction

Nicotinamide mononucleotide (NMN) is a direct precursor of nicotinamide adenine dinucleotide (NAD^+^), a vital coenzyme essential for cellular health and energy metabolism and healthy aging [1]. Notably, the aging process is characterized by a progressive reduction in cellular NAD^+^ levels. This decline in NAD^+^ levels is causally implicated in a multitude of aging-related diseases, including atherosclerosis, arthritis, hypertension, cancer and metabolic disorders [2]. Despite the paramount importance of NAD^+^ in promoting healthy aging and enhancing longevity, there are currently no directly effective supplementation means available due to its impermeable nature. Supplementation with NMN has emerged as a direct and highly effective strategy for elevating NAD^+^ levels, thereby mitigating aging-related physiological decline and metabolic disorders [3,4,5]. This highlights its substantial potential as a promising anti-aging nutraceutical for improving human health, driving notable interest across industries such as nutraceuticals, food additives, and cosmetics. The global market for NMN was valued at approximately US $98.5 million in 2019 and is projected to expand at a compound annual growth rate (CAGR) of 7.3% from 2021 to 2026 [6]. Given this rising demand, the production of NMN has become an important concern.

Currently, industrial production of NMN is primarily achieved through chemical synthesis [7]. However, the chemical process involves stringent reaction conditions and the use of toxic solvents, making it economically inviable and environmentally unsustainable [8]. In vitro enzymatic or biocatalytic conversion utilizes isolated enzymes (e.g., nicotinamide riboside kinase, NRK) to catalyze the conversion of substrates such as nicotinamide riboside (NR) and ATP into NMN representing a promising route characterized by high yield, rapid kinetics, high selectivity, and fewer by-products. This approach is also considered more environmentally sustainable and aligns with green manufacturing principles. For instance, Tang et al. achieved an NMN yield of 14.6 g/L by employing NRK for ATP- and NR-dependent conversion and optimizing the process through orthogonal design [9]. Despite these advances, the industrial competitiveness of this approach remains limited by several challenges, including low enzyme stability and activity, limited reusability, and the high cost associated with ATP consumption. In contrast, microbial fermentation-based strategies, particularly the construction of efficient microbial cell factories, provide a promising alternative for the cost-effective and sustainable industrial production of NMN, and have been gaining preference in recent years [10,11].

NMN biosynthesis occurs mainly through three pathways: (1) the de novo pathway, (2) the Preiss–Handler pathway and (3) the salvage pathway [12]. Characterized by its simplicity, low energy consumption, and the ready availability of nicotinamide (NAM), the NAM-based salvage pathway is the most engineered route and the leading strategy for achieving economical and high-level production of NMN [13,14]. This route involves the condensation reaction of NAM with 5′-phosphoribosyl-1-pyrophosphate (PRPP) catalyzed by nicotinamide phosphoribosyltransferase (NAMPT) (EC 2.4.2.12), resulting in the biosynthesis of NMN. As a co-substrate, the availability of PRPP, an intermediate in the pentose phosphate (PP) pathway, is essential for NMN synthesis [15]. Given that glucose catabolism is primarily initiated through glycolysis and the PP pathway, and only a fraction of glucose enters the PP pathway [16,17], the supply of PRPP is insufficient, constituting a major bottleneck for NMN production. Current strategies to increase PRPP availability mainly focus on two aspects. The first strategy involves enhancing or regulating the expression of genes in the PP pathway, such as overexpressing *zwf* and *gnd* to pull more glucose into the PP pathway [18,19,20,21]. For example, Shoji et al. enhanced the intracellular PRPP pool derived from glucose by overexpressing key genes in the PP pathway, achieving an extracellular NMN titer of up to 6.79 g/L [18]. The second strategy is to attenuate the glycolytic pathway by knocking out key genes, such as *pgi* and *pfkA*/*pfkB* or *pykA*/*pykF*, to redirect glucose towards the PP pathway [22,23]. For instance, Xiong et al. disrupted the Embden–Meyerhof–Parnas (EMP) pathway through deletion of *pfkA* and *talA*, resulting in an extracellular NMN titer of up to 20.13 g/L [24]. Despite significant advances in metabolic engineering, the intrinsic competition for central carbon metabolic fluxes continues to pose a critical bottleneck in the development of high-yield NMN-producing strains. Specifically, the excessive redirection of carbon flux toward NMN biosynthesis imposes substantial metabolic stress on host cells, triggering a cascade of cellular responses, including (1) activation of oxidative stress pathways, (2) disruption of redox homeostasis, and (3) reallocation of cellular resources away from biomass synthesis. These combined effects result in a reduction in the specific growth rate under industrial fermentation conditions, ultimately limiting NMN titers. Therefore, it is necessary to strike a balance between enhancing PRPP supply and maintaining robust cell growth for high level production of NMN. The co-utilization of carbon sources is considered an effective strategy to address the contradiction between cell growth and product synthesis [25,26,27,28]. As a major byproduct of biodiesel production, glycerol is particularly attractive due to its low cost and abundance, making it a promising co-substrate for cost-effective fermentation [29,30]. The co-utilization strategy of glucose and glycerol has been successfully applied to the biosynthesis of various compounds, such as myo-inositol [31], and N-Acetyl-glucosamine [32]. To date, it remains unclear whether the co-utilization strategy of glucose and glycerol can be applied to improve NMN biosynthesis through alleviating the trade-off between cell growth and product formation.

**Figure 1:**
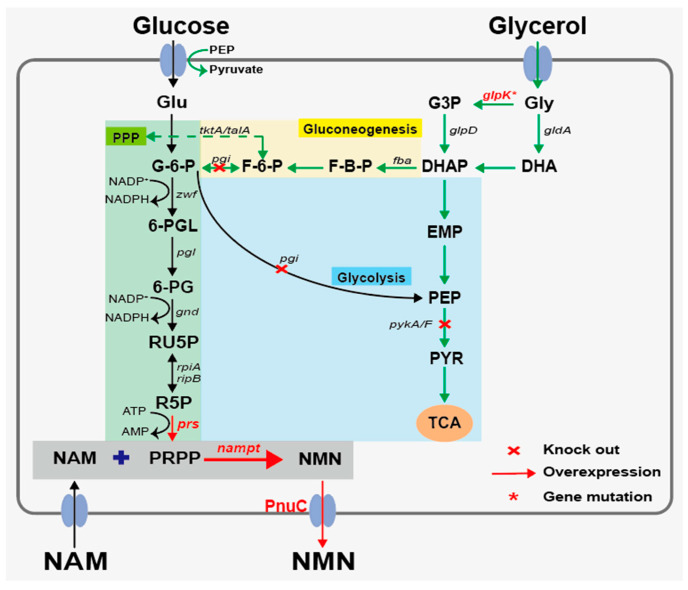
Strategy for co-utilization of mixed carbon sources of glucose and glycerol for NMN production. Red crosses indicate gene deletion; red arrows indicate gene overexpression; green-colored arrows indicate the glycerol metabolic pathway mechanism. The main metabolite abbreviations are as follows: G3P, glycerol 3-phosphate; DHAP, glycerone phosphate; EMP, Embden–Meyerhof–Parnas pathway; PEP, phosphoenolpyruvate; PYR, pyruvate; TCA, tricarboxylic acid cycle; G-6-P, glucose-6-phosphate; 6-PGL,6-phosphogluconolactone; 6-PG, 6-phosphogluconate; RU5P, ribulose-5-phosphate; R5P, ribose-5-phosphate; PRPP, phosphoribosylpyrophosphate. NAM, nicotinamide; PRPP, phosphoribosyl pyrophosphate; NMN, nicotinamide mononucleotide; PnuC, the transport of NMN outside the cell. Key gene abbreviations: *pgi*, phosphoglucose isomerase; *pykA*/*F*, encoding pyruvate kinases; glpK*, glycerol kinase gene; *prs*, phosphoribosyl pyrophosphate synthetase; and *nampt*, nicotinamide phosphoribosyltransferase.

Inspired by our previous work that co-utilization of xylose and glucose can enhance NMN production [33], we propose a novel strategy in which glucose is preferentially utilized for NMN biosynthesis, while glycerol is utilized as a secondary carbon source to support cell growth (Figure 1). Specifically, the key glycolytic genes (e.g., *pgi*, *pykA*/*pykF*) were deleted to disrupt glucose catabolism through the glycolytic pathway. Meanwhile, the gene associated with feedback inhibition was introduced, enabling glycerol to be utilized in the presence of glucose. The resulting strain GN02 achieved an NMN titer of 32.92 g/L after 72 h of fed-batch fermentation in a 2 L bioreactor. This study offers an effective strategy for the high-level production of NMN and its derivatives, and lays a foundation for industrial-scale production of NMN.

## 2. Materials and Methods

### 2.1. Materials, Strains and Plasmids

The strains and plasmids used in this study were listed in Table 1. *E. coli* Trelief™ 5α (Tsingke Company, Beijing, China) was used for DNA cloning; the previously reported NMN-producing strain NMN008, carrying two plasmids pBAD-VniNampt-BsPrs and pRSF-BmPnuC, was used as the host bacteria for genetic engineering [33]. The pCas9 and pTarget plasmids were obtained from our laboratory collection for gene editing. High-fidelity DNA polymerase (2× High Fidelity Master Mix) was purchased from Vazyme (Beijing, China). Plasmid extraction and gel purification kits were purchased from Omega (Beijing, China). Standards of glucose, glycerol, NMN, NAM, and other chemicals were purchased from Sigma-Aldrich (Shanghai, China).

### 2.2. CRISPR/Cas9-Based Genetic Editing Technology

Gene knock-in and knock-out experiments were conducted with the CRISPR-Cas9 two-plasmid system [34]. N20 sequences were predicted using an online tool (http://crispor.tefor.net/crispor.py, accessed on 19 February 2024) to construct corresponding pTarget plasmids. Taking *pgi* knockout as an example, first, the upstream and downstream homologous arms (500 bp) were amplified using pgi-up-F/pgi-up-R and pgi-down-F/pgi-down-R respectively, and then, the upstream and downstream fragments were connected by overlapping PCR using pgi-up-F/pgi-down-R to obtain the donor double-stranded DNA (dsDNA). Subsequently, the donor DNA (800 ng), along with a pTarget-pgi plasmid (200 ng), was co-transformed into the electro-competent host cells carrying the pCas9 plasmid using Eppendorf Eporator (Hamburg, Germany) at 2.5 kV. The transformed cells were cultivated on LB agar plates containing kanamycin (50 μg/mL) and spectinomycin (50 μg/mL) at 30 °C for 20 h. The correct transformants were identified using primers pgi-JD-F/pgi-JD-R and confirmed by sequencing. The verified strains were subsequently cultivated in LB medium supplemented with 2 g/L of L-arabinose at 30 °C for 20 h to cure the target gRNA-carrying plasmid. The pCas9 plasmid was subsequently cured through cultivation at 42 °C for 24 h. All primers for gene editing used in this study were listed in the Supplementary Material (Table S1).

### 2.3. Media and Culture Conditions

*E. coli* strains were routinely cultivated at 37 °C and 220 rpm in Luria–Bertani (LB) medium (5 g/L yeast extract, 10 g/L tryptone, 10 g/L NaCl). For characterization and fermentation of engineered strains, a defined R medium was used, which contains (per liter) 8 g of (NH_4_)_2_HPO_4_, 13.3 g of KH_2_PO_4_, 1.2 g of MgSO_4_·7H_2_O, 1.7 g of citric acid, and 10 mL of a trace metal solution. The trace metal solution (prepared in 1 L of 5 M HCl) consisted of 10 g of FeSO_4_·7H_2_O, 2.25 g of ZnSO_4_·7H_2_O, 1 g of CuSO_4_·5H_2_O, 0.5 g of MnSO_4_·5H_2_O, 0.23 g of Na_2_B_4_O_7_·10H_2_O, 2 g of CaCl_2_·2H_2_O and 0.1 g of (NH_4_)_6_Mo_7_O_24_. Glucose and glycerol were used as carbon sources, and their concentrations were adjusted according to the experimental design. The cultivation was carried out at 30 °C and 220 rpm in a shake flask. When necessary, the medium was supplemented with 100 μg/mL ampicillin, 50 μg/mL kanamycin, 2 g/L L-arabinose, and 2 g/L lactose.

### 2.4. Whole-Cell Biocatalysis of NMN

The engineered strains were pre-cultivated in LB medium in 15 mL test tubes at 37 °C and 220 rpm. The overnight cultures were inoculated at a ratio of 1% (*v*/*v*) into 50 mL of fresh LB medium at 200 rpm at 37 °C, and then induced with L-arabinose and lactose at an OD_600_ of 0.8. The induced cultures were at 30 °C and 220 rpm for 24 h. For whole-cell biocatalysis, 200 OD cells were collected by centrifugation at 4 °C and 5000× *g* for 10 min. The pellets were resuspended into 20 mL of R medium (100 mL flask) containing 3 g/L NAM and different carbon sources.

The sample was centrifuged at 10,000× *g* for 5 min, and the supernatant was collected for further extracellular concentration measurement. The concentrations of NAM and NMN were determined using high-performance liquid chromatography (HPLC).

### 2.5. Fed-Batch Cultivation

The seed culture was prepared in 100 mL of LB medium and cultivated at 37 °C with shaking at 220 rpm for 12 h. The culture was then inoculated into a 2 L bioreactor containing 900 mL of initial R media supplemented with 2.5 g/L glucose and 2.5 g/L glycerol, and cultivated at 37 °C. When the glucose and glycerol in the initial medium were about to be depleted, a feeding solution composed of 50% glucose and 50% glycerol (*w*/*w*, 1:1) was added at a rate of 2 g/L/h. When the OD_600_ reached approximately 30 (around12 h), L-arabinose and lactose were added to induce protein expression, and the temperature was set at 30 °C. When the OD_600_ reached approximately 50 (around 24 h), the substrate NAM was added at a rate of 0.4–0.6 g/L/h. The pH was maintained at 7.0 by automatic addition of NH_4_OH (50%, *v*/*v*), while the dissolved oxygen (DO) level was controlled at 30% of air saturation via adjustment of agitation speed and aeration rate. When necessary, the medium was supplemented with 100 μg/mL ampicillin, 50 μg/mL kanamycin.

### 2.6. Analytical Methods

Cell growth was monitored by measuring the optical density at 600 nm (OD_600_) using a UV-Vis spectrophotometer purchased from Thermo Fisher Scientific Inc. (Waltham, MA, USA). Dry cell weight (DCW) was calculated based on the measured OD600, as follows: 1 DCW [g/L] = 0.32 × OD600 + 0.02 [35].

NMN and its analogs were detected using an HPLC system equipped with a ZORBAX SB-AQ Stable Bond analytical column (5 µm, 4.6 × 250 mm; Agilent Technologies, Santa Clara, CA, USA). The flow rate was set at 1.0 mL/min and the column temperature was maintained at 30 °C. The injection volume and detector wavelength were set to 10 μL and 263 nm, respectively. Mobile phase A was a 0.1% (*v*/*v*) aqueous trifluoroacetic acid solution, and mobile phase B was acetonitrile. The elution conditions were as follows: 0–5 min, gradient increase from 0% to 5% acetonitrile; 5–10 min, gradient increase from 5% to 20% acetonitrile; 10–17 min, gradient increase from 20% to 100% acetonitrile; 17–20 min, maintenance of 100% acetonitrile; 20–25 min, gradient decrease from 100% to 0% acetonitrile; and 25–30 min, maintenance of 0% acetonitrile.

Glucose and glycerol were measured using an HPLC system equipped with a Bio-Rad, Hercules, CA, USA, Aminex HPX-87H column (7.8 mm × 300 mm; Hercules, CA, USA) and a refractive index detector. The column temperature was set at 30 °C, and the mobile phase consisted of 5 mM H_2_SO_4_ at a flow rate of 0.6 mL/min.

## 3. Results and Discussion

### 3.1. Enhancing NMN Biosynthesis by Weakening Glucose Consumption via Glycolysis

Glucose metabolism is primarily accomplished through glycolysis and the PP pathway. There is a trade-off relationship between the two pathways. Thus, we speculated that weakening or disrupting the glycolytic pathway would probably redirect more glucose toward the PP pathway, thereby increasing the supply of precursor PRPP and boosting NMN production.

**Figure 2:**
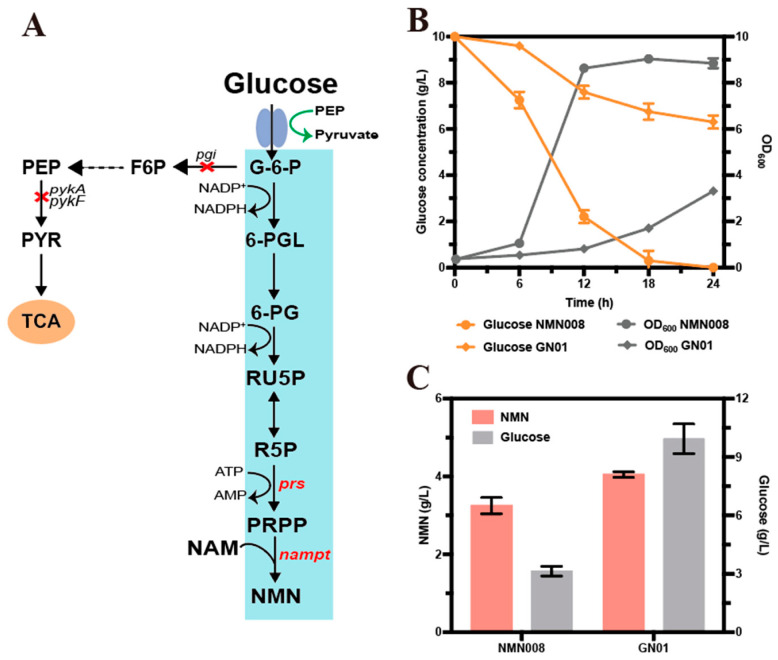
Effects of the disruption glycolytic genes (*pgi* and *pykA*/*pykF*) on strain growth, glucose consumption, and NMN production. (**A**) Overview of the carbon metabolism and the target genes being deleted. (**B**) Cell growth and glucose consumption in shake flask. (**C**) Whole-cell catalysis of NMN synthesis and residual glucose. Data are presented as mean ± s.d. (*n* = 3 biological replicates).

To test this speculation, we sequentially knocked out the glycolytic genes *pgi* (encoding phosphoglucose isomerase, gene ID:948535), *pykA* (encoding pyruvate kinase I, gene ID:946180) and *pykF* (encoding pyruvate kinase II, gene ID:946179) in strain NMN008 (Figure 2A), generating strain GN01 (NMN008Δ*pgi*Δ*pykA*Δ*pykF*). As expected, deletion of the key glycolytic genes (*pgi*, *pykA*, and *pykF)* effectively redirected glucose metabolism and improved NMN production efficiency. After 24 h of whole-cell catalysis in R medium, the engineered strain GN01 yielded 4.05 g/L NMN with a glucose consumption of 5.17 g/L, whereas the control strain NMN008 generated 3.25 g/L NMN at a glucose cost of 11.87 g/L (Figure 2C). These results implied that weakening the EMP glycolytic pathway may redirect more carbon flux toward the PP pathway, potentially increasing the availability of the precursor PRPP for NMN biosynthesis. However, disruption of the glycolytic pathway exerted a detrimental effect on cellular growth, the OD_600_ value of strain GN01 (OD_600_ = 3.31) was reduced by 61.51% relative to that of strain NMN008 (OD_600_ = 8.6) (Figure 2B). This pronounced growth defect presents a major limitation for large-scale fermentation applications.

### 3.2. Enhancing NMN Biosynthesis via Co-Utilization of Glucose and Glycerol

To overcome the growth defect associated with impaired glycolysis, a second carbon source (e.g., glycerol) was supplemented to support cellular growth, while glucose was used as a substrate to maintain the rerouted metabolic flux toward NMN biosynthesis. To enhance glycerol metabolism in the presence of glucose, a variant of glycerol kinase GlpK* (GlpK^G913A^), previously reported to effectively relieve glucose-induced carbon catabolite repression and enhance glycerol utilization [36,37], was introduced to replace native *glpK* in GN01, generating strain GN02.

**Figure 3:**
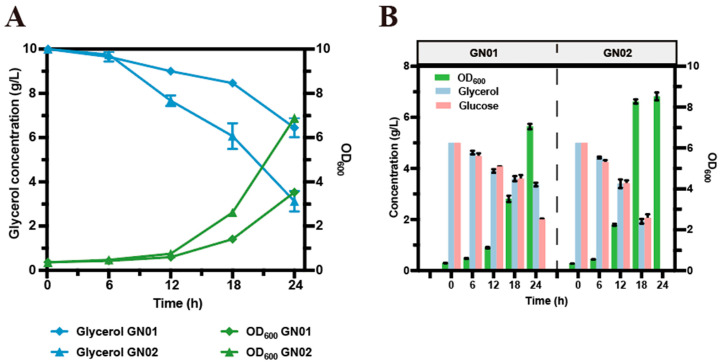
Cell growth and sugar consumption of the engineered strains. (**A**) Cell growth and glycerol consumption in shake flasks using glycerol as the sole carbon source. (**B**) Cell growth and residual glucose and glycerol in shake flasks using mixed carbon source of glycerol and glucose. Data are presented as mean ± s.d. (n = 3 biological replicates).

As shown in Figure 3A, strain GN01 reached an OD_600_ of only 3.53 and consumed 3.55 g of glycerol when glycerol was used as the sole carbon source. In contrast, the strain expressing the feedback-resistant glpK* (GN02) exhibited significantly improved growth (OD_600_ = 6.88) and consumed 6.87 g of glycerol under the same conditions, representing a 93.52% increase in glycerol consumption. Under mixed carbon sources, strain GN02 also showed markedly enhanced growth compared with GN01 (Figure 3B). These results indicated that the introduction of glpK* improved cell growth and was consistent with enhanced glycerol utilization when glycerol was used as the sole carbon source (Table S2) or in a mixture of glucose and glycerol (Table S3). This genetic modification, in turn, led to increased consumption of glucose.

**Figure 4:**
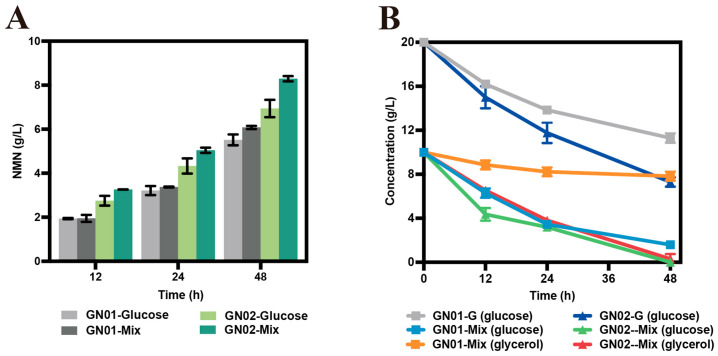
Whole-cell catalysis of NMN synthesis using engineered strains. (**A**) Comparison of NMN production in GN01 and GN02 strains with different carbon sources. (**B**) Residual glucose and glycerol. Mix indicated that glucose and glycerol were used as a mixed carbon source. Data are presented as mean ± s.d. (*n* = 3 biological replicates).

Having examined the effect of carbon source composition on cell growth, we next analyzed the effect of these genetic modifications on NMN production; we compared the performance of strains GN01 and GN02 under different culture conditions. Extracellular NMN was undetectable in the engineered strains when glycerol was used as the sole carbon source. With glucose as the sole carbon source, the engineered strain GN02 produced 6.94 g/L NMN. Notably, a higher titer of 8.30 g/L was achieved under glucose–glycerol mixed carbon source conditions at a 1:1 (*w*/*w*) ratio, where the strain exhibited the best growth performance (Figure S1). By comparison, the strain GN01 reached only 5.52 g/L and 6.08 g/L NMN under the same respective conditions (Figure 4). These results demonstrated that the introduction of glpK* facilitated the co-utilization of glucose and glycerol and concomitantly improved NMN biosynthesis, highlighting carbon source co-utilization as a robust strategy to enhance whole-cell catalytic performance and mitigate the growth-production trade-off in engineered strains.

### 3.3. NMN Production via High-Cell-Density Fermentation

**Figure 5:**
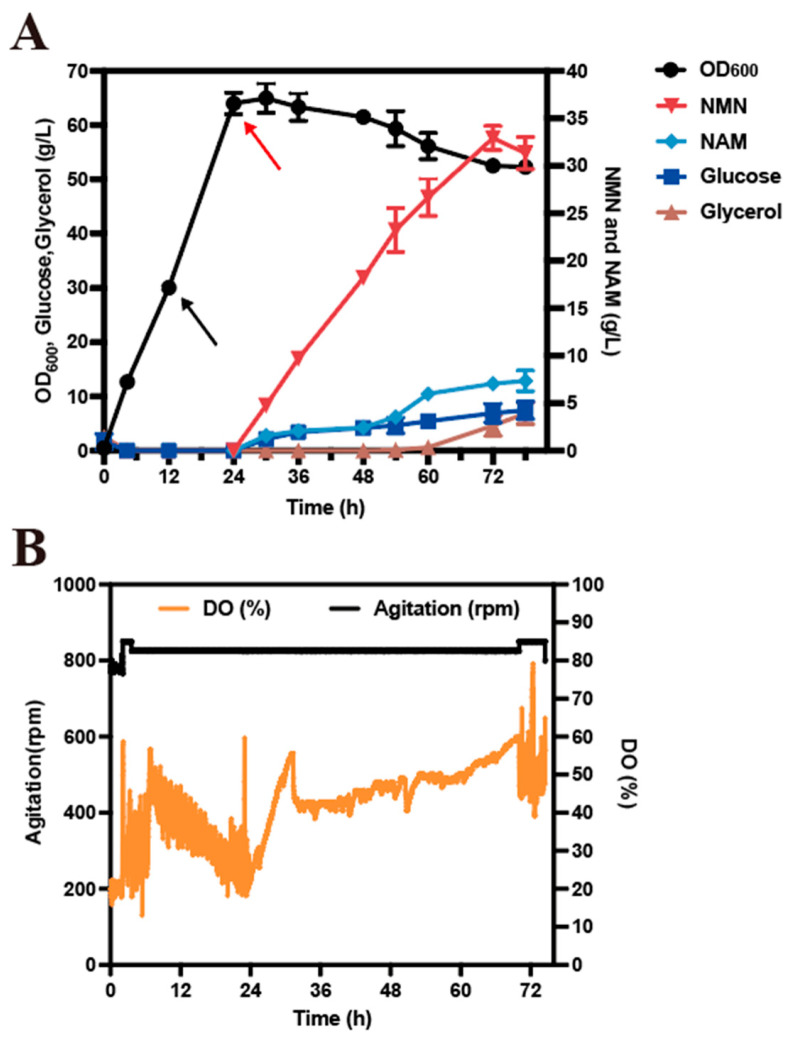
Fed-batch production of GN02 in 2 L bioreactors. (**A**) NMN production, residual nicotinamide, cell growth (OD_600_), and residual concentrations of glucose and glycerol during fermentation. (**B**) Relationship between dissolved oxygen (DO) and agitation speed during fermentation. Time 0 h represents the start of fermentation. The black arrows represent the addition of L-arabinose and lactose at 12 h to induce protein expression. The red arrows indicate the initiation of substrate (NAM) feeding at 24 h, marking the start of the bioconversion phase. Data are presented as mean ± s.d. (*n* = 3 biological replicates).

To evaluate the potential of the engineered strain GN02 for the industrial production of NMN, high-cell-density fermentation was conducted in a 2 L bioreactor using a fed-batch strategy. The mixed carbon source of glucose and glycerol was fed at a rate of 2 g/L/h, while NAM was continuously supplied at a rate of 0.4–0.6 g/L/h throughout the fermentation process. The resulting strain GN02 produced 32.92 g/L of NMN after 72 h, with a yield of 0.80 g/g glucose and 0.39 g/g total carbon, and maintained a stable biomass level throughout the whole-cell bioconversion process, accompanied by a relatively stable dissolved oxygen (DO) profile (Figure 5). In contrast, for strain NMN008, in our previous work using glucose as the sole carbon source, a maximum NMN titer of 26.07 g/L and a yield of approximately 0.29 g/g glucose were obtained, and the engineered strain exhibited a pronounced decline in cell density during the NMN production phase [33], which was accompanied with a sharp increase in DO (Figure S2). Given that DO profiles serve as a real-time indicator of cellular respiration and physiological status, these observations indicate that glucose–glycerol co-utilization effectively enhanced respiratory activity and growth stability, thereby improving NMN biosynthesis. This work demonstrated the effectiveness of a carbon partitioning strategy in which glucose is primarily directed toward PRPP synthesis, while glycerol supports cellular growth.

## 4. Conclusions

This research shows that the co-utilization of glucose and glycerol is feasible for alleviating the growth–production trade-off in NMN biosynthesis. Our work offers a promising strategy to improve NMN production, and also sheds light on enhancing the production of related chemicals derived from PP pathway-driven bioprocesses. Although the engineered strains exhibited improved NMN production, the underlying metabolic mechanisms were not fully elucidated in this study. In particular, intracellular metabolite levels and carbon flux distribution between glycolysis and the pentose phosphate pathway were not directly quantified. Future studies will focus on integrated metabolomic and ^13^C metabolic flux analysis to better characterize carbon flux redistribution patterns and thereby provide quantitative guidance for iterative optimization of NMN biosynthetic efficiency.

## Figures and Tables

**Table 1:** Strains and plasmids used in this study.

Strains	Characteristics	Source
*E. coli* Trelief™ 5α	F^−^φ80(*lacZ*)Δ*M15*Δ (*lacZYA-argF*) U169 *deoR endA1 recA1 hsdR17* (rk^−^, mk^+^) *supE44λ-thi-1gyrA96 relA1 phoA*	Tsingke company (Beijing, China)
*E. coli* BW25113	*rrnB3*Δ*lacZ4787 hsdR514*Δ(*araBAD*)*567*Δ(*rhaBAD*)*568rph-1*	Invitrogen (Carlsbad, CA, USA)
BA04	BW25113Δ*pncC*Δ*nadR*Δ*ushA*Δ*pncA*Δ*umpG*Δ*umpH*	[33]
NMN008	pBAD-VniNampt-BsPrs and pRSF-BmPnuC in BA04	[33]
GN01	NMN008Δ*pgi*Δ*pykA*Δ*pykF*	This work
GN02	GN01Δ*glpk*::glpk*(G913A)	This work
pTarget	20 bp sgRNA, Spec^R^	Laboratory storage
pCas9	repA101(Ts) Kan Pcas-cas9 ParaB-Red lacIq Ptrc-sgRNA-pMB1	Laboratory storage
pTarget-pgi	pTarget with sgRNA for knockout of *pgi*	This work
pTarget-pykA	pTarget with sgRNA for knockout of *pykA*	This work
pTarget-pykF	pTarget with sgRNA for knockout of *pykF*	This work
pTarget-glpk	pTarget with sgRNA for knockout of *glpk*	This work

## Data Availability

The original contributions presented in this study are included in the article/Supplementary Material. Further inquiries can be directed to the corresponding authors.

## Supplementary Materials

The following supporting information can be downloaded at https://www.mdpi.com/article/10.3390/microorganisms14040897/s1: Table S1: Primers used for gene editing; Table S2. Comparison of glycerol consumption rates between GN02 and GN03 strains (glycerol as sole carbon source); Table S3. Glucose and glycerol consumption rates of GN02 and GN03 strains under mixed carbon source conditions; Figure S1: Effects of different glucose-to-glycerol mass ratios (1:0, 4:1, 2:1, 1:1, 1:2, 1:4, and 0:1) on the growth of strain GN02 at a total carbon source concentration of 10 g/L; Figure S2: Fed-batch production of NMN008 in 2 L bioreactors.
