# Supplementary material for: High-Level Production of NMN in Escherichia coli Through Co-Utilization of Glucose and Glycerol
Source: Microorganisms. 2026 Apr 16;14(4):897. doi: 10.3390/microorganisms14040897 (PMC13119108; doi:10.3390/microorganisms14040897)
Supplement: Supplementary file 1 [file microorganisms-14-00897-s001.zip › microorganisms-4203142-supplementary.pdf]

High-Level Production of NMN in *Escherichia coli* Through Co-Utilization of Glucose and Glycerol

Jiajia Gan [1,2,†], Xiuzhen Chen [1,†], Yongzhi He [1], Yanfeng Zhang [3], Jin Zhong [1,2,*] and Zhiyang Dong [1,*]

1 State Key Laboratory of Microbial Diversity and Innovative Utilization, Institute of Microbiology, Chinese Academy of Sciences, Beijing 100101, China; ganjiajia@im.ac.cn (J.G.); chenxiuzhen@im.ac.cn (X.C.); heyz@im.ac.cn (Y.H.)

2 School of Life Science, University of Chinese Academy of Sciences, Beijing 100049, China

3 Shenzhen Siyomicro Bio-Tech Co., Ltd., Shenzhen 518100, China; zhangyf@siyobio.com

* Correspondence: zhongj@im.ac.cn (J.Z.); dongzy@im.ac.cn (Z.D.)

† These authors contributed equally to this work.

Table S1. Primers used for gene editing.

Name	Sequences (5'-3')
gRNA-pgi-F	taccagctgatccaccagttttagagctagaaatagca
gRNA-pgi-R	tggtggatcagctggtagaaactagtattatacctaggact
gRNA-pykA-F	cgttcagttcgacaatggttttagagctagaaatagca
gRNA-pykA-R	cattgtcgaactgaacgtccactagtattatacctaggact
gRNA-pykF-F	ccgactcgcgcagaagcgttttagagctagaaatagca
gRNA-pykF-R	gcttctgcgcgagtcgggcgactagtattatacctaggact
gRNA-glpK-F	gtgaactatgcgttggagttttagagctagaaatagca
gRNA-glpK-R	tccaacgcatagttcacttcactagtattatacctaggact
pgi-up-F	catcagaaagcacaatatcagcgc
pgi-up-R	tagcaatactcttctgattttgagaattgtga
pgi-down-F	aaatcagaagagtattgctatcatcgtcgatatgtaggccg
pgi-down-R	cgcaagcgcagatatggcaaa
pykA-up-F	cccacgatgcgatgaataagttctt
pykA-up-R	gtaatactccgttgactgaaacaaccag
pykA-down-F	ttcagtcaacggagtattacgtacgttgccggatgcgg
pykA-down-R	gattatgtctggaacacggtgcg
pykF-up-F	ataacaatatgctttggttcctgcc
pykF-up-R	attaattcacaaaagcaatagacagtcttagtctttaagttgagaaggatg
pykF-down-F	tattgcttttgtgaattaatttgtatatcgaagcg
pykF-down-R	cagtagagtcagcgcagaatg
glpk-up-F	atcgtttcacaagttgccggc
glpk*-up-R	accgcactttccaacgcatagttcacttcg
glpk*-down-F	atgcgttggaaagtgcggtg
glpk-down-R	ggcaaaccgttgagcgaactg
pgi-JD-F	ccaggttccagtccttcagct
pgi-JD-R	gtattagaaccatcaccgccaccg
pykA-JD-F	cattcactggataaatctgccatcaacc
pykA-JD-R	ccgtcgatattcctgccatgctg
pykF-JD-F	ctgcgctgaatgcgtttattgg
pykF-JD-R	cgataaagcgttgtatcgtcggg
glpk-JD-F	ctttctgtgctgcggctttagtt
glpk-JD-R	aattctcacctgtatgccagacagc
sp-JD-F	accgcctttgagtgagct
sp-JD-R	atcagaggtagttggcgtcatcg

Table S2. Comparison of glycerol consumption rates between GN02 and GN03 strains (glycerol as sole carbon source).

Strains	Time interval (h)	Volumetric consumption rate (g $L^{-1}h^{-1}$)	Specific consumption rate (g g^{-1}DCW h^{-1})
GN02	0-12	0.09	0.52
	12-24	0.21	0.31
GN03	0-12	0.19	0.98
	12-24	0.38	0.31

Table S3. Glucose and glycerol consumption rates of GN02 and GN03 strains under mixed carbon source conditions.

Strains	Substrate	0-12 h		12-24 h	
		Volumetric consumption rate (g $L^{-1}h^{-1}$)	Specific consumption rate (g g^{-1}DCW h^{-1})	Volumetric consumption rate (g $L^{-1}h^{-1}$)	Specific consumption rate (g g^{-1}DCW h^{-1})
GN02	Glucose	0.08	0.30	0.17	0.13
	Glycerol	0.09	0.35	0.04	0.03
GN03	Glucose	0.12	0.27	0.30	0.17
	Glycerol	0.13	0.31	0.28	0.16

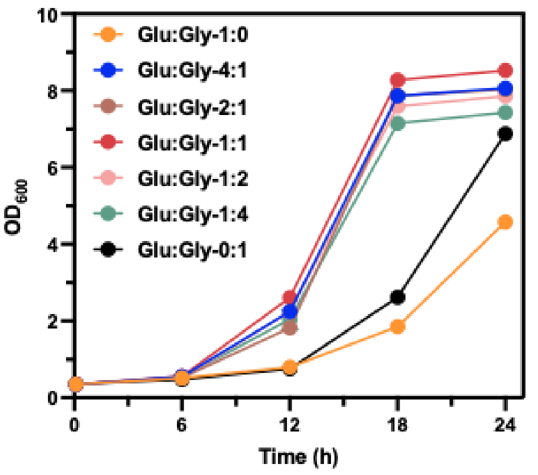

Figure S1. Effects of different glucose-to-glycerol mass ratios (1:0, 4:1, 2:1, 1:1, 1:2, 1:4, and 0:1) on the growth of strain GN02 at a total carbon source concentration of 10 g/L. Data are presented as mean ± s.d. (n = 3 biological replicates).

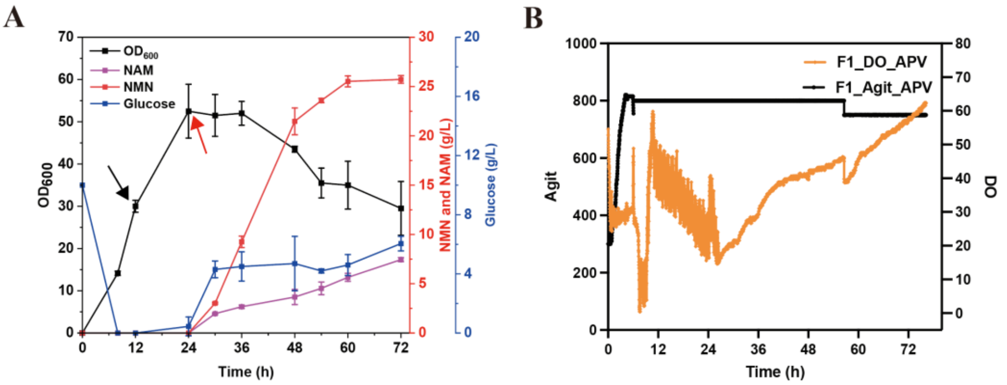

Figure S2. Fed-batch production of NMN008 in 2 L bioreactors. (A) NMN production, residual nicotinamide, cell growth (OD_{600}), and consumption of glucose during fermentation. (B) Relationship between dissolved oxygen (DO) and agitation speed. The black arrows represent the addition of arabinose and lactose to induce protein expression. The red arrows represent the start of the conversion. Data are presented as mean ± s.d. (n = 3 biological replicates).
